# People are at least as good at optimizing reward rate under equivalent fixed-trial compared to fixed-time conditions

**DOI:** 10.3758/s13423-025-02680-y

**Published:** 2025-04-03

**Authors:** Grant J. Taylor, Scott D. Brown, Nathan J. Evans

**Affiliations:** 1 School of Psychology, University of Queensland, Brisbane, Australia; 2 School of Psychology, University of Newcastle, Newcastle, Australia; 3 Department of Psychology, University of Liverpool, Liverpool, UK

**Keywords:** Evidence accumulation models, Reward rate, Optimal decision-making, Response time models

## Abstract

Finding an optimal decision-making strategy requires a careful balance between the competing demands of accuracy and urgency. In experimental settings, researchers are typically interested in whether people can optimise this trade-off, typically operationalised as reward rate, with evidence accumulation models serving as the key framework to determine whether people are performing optimally. However, recent studies have suggested that inferences about optimality can be highly dependent on the task design, meaning that inferences about whether people can achieve optimality may not generalise across contexts. Here, we investigate one typically overlooked design factor: whether participants spend a fixed amount of time on each block (fixed time) or have a fixed number of trials in each block (fixed trials). While fixed-time designs are typically thought to be the most appropriate for optimality studies, as to maximise the number of correct responses participants must optimise RR, our Experiments 1 and 2 indicate that people are at least as good at optimising reward rate under fixed-trial designs as fixed-time designs. However, Experiment 3 provides some evidence that fixed-trial designs with no instructions may not be at least as good as fixed-time designs with very specific instructions. Importantly, these findings challenge the idea that fixed-time designs are the most appropriate for reward rate optimality studies, and further emphasise the importance of carefully considering study design factors when making inferences about optimality in decision-making.

**Supplementary Information:**

The online version contains supplementary material available at 10.3758/s13423-025-02680-y.

## Introduction

An optimal decision-making strategy in natural settings balances the competing demands of accuracy and urgency in relation to the goals, motivations and resources of the decider (Anderson, 1991; Khodadadi et al., 2014; van Ravenzwaaij et al., 2012). For an individual who wants to be accurate and has plenty of time to achieve this goal, being cautious would be the optimal strategy, as more urgent strategies would unnecessarily reduce accuracy. However, for another individual, being fast and less accurate may be optimal given their unique circumstances. In experimental paradigms, one way to avoid the subjectivity and variability inherent in individual goals – which in turn, influence the optimal strategy – is to define optimal decision-making in terms of maximising reward rate. From this perspective, optimality can be described as the best way to balance the trade-off between the speed and accuracy of a decision, with strategies that are too cautious/urgent, producing non-optimal outcomes.

Reward rate (RR), typically defined as the rate at which correct decisions are produced, is a metric commonly used in the evidence accumulation modelling (EAM) literature when investigating optimal decision-making (Bogacz et al., 2006; Simen et al., 2009; Bogacz et al., 2010; Drugowitsch et al., 2015; Evans & Brown, 2017). EAMs are the dominant framework for rapid decision-making, with their basic assumption being that evidence is accumulated for the different decision options, until sufficient evidence is reached for one of the options, and a response is triggered. EAMs are able to inform researchers about the decision process by analysing choice response time data to reveal latent variables, such as decision-making ability relative to task difficulty (drift rate, *v*), decision boundary where responses are triggered (threshold, *a*), response biases (*z*), and encoding/motor response time (*ter*) (see Ratcliff et al., 2016 and Evans & Wagenmakers, 2020 for reviews). A cautious decider would set their threshold higher, accumulating more information to ensure a better chance of a correct decision, and subsequently take longer to make their decision. In contrast, a less cautious decider would set a lower threshold, not take as long to choose, but would produce fewer correct decisions.

In the context of optimal decision-making, EAMs are able to estimate not only how cautious a person is in completing a task, but also how cautious they should have been in order to maximise their RR. However, previous research has come to contrasting conclusions about the ability of humans to adopt RR optimal strategies (Simen et al., 2009; Evans & Brown, 2017; Evans et al., 2019; Balci et al., 2011; Starns & Ratcliff, 2010, 2012; Hawkins et al., 2012; Evans & Hawkins, 2019; Evans et al., 2020; Trueblood et al., 2020; Drugowitsch et al., 2012). For example, Simen et al. (2009) showed that participants could adapt their speed–accuracy trade-off and response biases when subject to changing task conditions, to maximise RR and attain optimal or near optimal thresholds. In contrast, while Evans and Brown (2017) showed that people can become more optimal in their decision-making when provided practice and feedback, their general findings still supported the notion that caution is the default behaviour, though subsequent research by Evans et al. (2019) suggested that default behaviour is approximately optimal under conditions with longer timeouts after errors. Other studies have also suggested people become sub-optimally cautious as task difficulty increases (Balci et al., 2011; Starns & Ratcliff, 2012), showing a qualitative disagreement with optimal behaviour. These conflicting results have somewhat limited general conclusions about whether humans are able to – and if so, whether they choose to Hawkins et al. (2012); Starns and Ratcliff (2012) – adopt RR optimal decision strategies.

One explanation for some^1^ of these conflicting findings is that participants’ ability to achieve optimality may be heavily dependent on the task design (Evans et al., 2019; Simen et al., 2009). Specifically, the optimal level of caution for a given situation depends on a large number of factors outside of the participant’s direct control. For example, given all other factors are equal, participants who are worse at the task (i.e., with lower drift rates) should be more urgent than those who are better at the task, as there is little benefit from sampling more information due to the low quality (Bogacz et al., 2006). The pace of the task – often operationalised as the response-stimulus interval (RSI), the time between the participant’s response and the stimulus presentation in the next trial – also influences the optimal strategy, as slower tasks designs mean that a lot of time is already being wasted regardless of participant speed, making being more cautious optimal (Simen et al., 2009). More generally, any factor that can influence the total task time or the ability to gain rewards can influence the precise optimal strategy, meaning that participants may be better able to achieve optimality in circumstances where the task optimality demands align with default strategies. In line with this explanation, Evans et al. (2019) were able to create a task design where on average participants were in line with the optimal strategy, suggesting that task design elements may explain some of the previous conflicting findings in RR optimality research.

**Table 1:** Comparisons of study designs as the basis for Experiments 1, 2 and 3

Study	Fixed trial	Fixed time	Instructions	Feedback
	(per block)			
**Experiment** 1				
Random Dot Motion Task			2 levels:	1 level:
20 Blocks per participant	40 Trials	60 s	Reward rate	Medium only
Difficulty - 1 level (10% coherence)			None	
**Experiment** 2				
Random Dot Motion Task			2 levels:	1 level:
20 Blocks per participant	40 Trials	60 s	Reward rate	Low only
Difficulty - 1 level (10% coherence)			None	
**Experiment** 3				
Random Dot Motion Task			2 levels:	1 level:
20 Blocks per participant	40 Trials	60 s	None (fixed trial only)	Medium only
Difficulty - 1 level (10% coherence)			Reward rate (fixed time only)	
**SR2012**				
Numerical discrimination Task			2 levels	2 levels:
2 X 30 Blocks per participant	40 Trials	30 s	None (fixed trial only)	Low (fixed trial only)
Difficulty – 3 levels			Reward rate (fixed time only)	Medium (fixed time only)
**EB2017**				
Random Dot Motion Task				3 levels:
24 Blocks per participant	40 Trials	60 s	1 level:	Low
Difficulty - 1 level (10% coherence)			Reward rate	Medium
				High

**SR2012** - Starns & Ratcliff, 2012; **EB2017** - Evans & Brown, 2017 Reward rate - Explicit instructions to promote reward rate behaviour; None - No explicit instructions given Low - trial-by-trial feedback; Medium - includes Low plus block-by-block feedback High - same as Medium feedback, but with extra guidance on how to improve Experiment 3 has modified reward rate instructions and error message time delay set to 0 (see text for details)

One important task design factor, which has received limited empirical investigation, is whether participants spend a fixed amount of time on each block – meaning that the number of trials they complete in each block is variable (i.e., fixed-time design) – or complete a fixed number of trials in each block – meaning that the time spent on each block is variable (i.e., fixed-trial design). Importantly, fixed-time designs create a situation where the amount of time spent on the task (i.e., the overall “rate” at which people are able to obtain rewards) is constant, and participants are able to complete more trials to gain more chances at correct responses by performing faster, making that the optimal strategies for maximizing the total number of rewards and maximizing the RR are identical. Fixed-time designs are also common in non-human primate decision-making studies (e.g., Roitman & Shadlen, 2002), which have often concluded that non-human primates behave in a near-optimal manner (Hawkins et al., 2015). In contrast, fixed-trial designs allow participants to spend as long as they like on the task, creating a situation where maximizing the total number of rewards requires an extremely cautious strategy, whereas maximizing RR requires a careful balancing of speed and accuracy.

The natural requirement in fixed-time designs of carefully balancing speed and accuracy to maximise the total number of rewards, as opposed to fixed-trial designs where these goals are competing, has led to several studies exclusively using fixed-time conditions (Balci et al., 2011; Bogacz et al., 2010; Simen et al., 2009), showcasing an assumption in the literature that fixed-time designs are better suited than fixed-trial designs to studying RR optimality.

However, in the limited number of experiments that have included both fixed-time and fixed-trial conditions (see Table 1), the findings have been mixed. In line with the assumption that people are better at optimizing RR under fixed-time conditions, Starns and Ratcliff (2012) found that while participants were generally sub-optimal – both quantitatively and qualitatively when faced with a difficulty manipulation – those who performed the task under fixed-time conditions were generally closer to optimality than those who performed the task under fixed-trial conditions. However, as the goal of Starns and Ratcliff (2012) was to assess age-related differences in optimality, and both fixed-time and fixed-trial conditions were largely present to ensure the robustness of the core results, they did not attempt to make these conditions as equivalent as possible, or make any strong conclusions about differences between the designs. For example, while (Starns & Ratcliff, 2012) explicitly instructed those in the fixed-time condition to try and achieve RR optimality, with feedback about the number of correct responses provided after each block, this was not the case for those in the fixed-trial condition, who performed the task with limited experimenter input. In contrast to these results, Evans and Brown (2017) consistently found participants to be closer to optimality under fixed-trial conditions than fixed-time conditions, with the instructions and feedback in both conditions design to be as equivalent as possible in encouraging participants to achieve RR optimality. Importantly, these conflicting findings make it unclear whether – and if so, how – the task design factor of fixed-time/trials influences participant’s ability to achieve optimality.

The current study aims to provide a more comprehensive investigation into whether people behave more optimally in fixed-time designs compared to fixed-trial designs. Specifically, the current study focuses on assessing two of the key experimental design factors that differed between the studies of Starns and Ratcliff (2012) and Evans and Brown (2017); the instructions and feedback given to participants (see Table 1). Specifically, Experiment 1 manipulates both whether participants are placed under fixed-time or fixed-trial conditions, as well as whether participants are given no specific instructions about their performance goal in the task or told to maximise RR, with all participants receiving feedback after each block about their accuracy, response time, and RR. Experiment 2 uses the same manipulations as Experiment 1, but participants are not given any information about RR after blocks. Finally, Experiment 3 attempts to replicate the instruction differences between the fixed-time and fixed-trial conditions from Starns and Ratcliff (2012) – as well as the lack of error timeouts in the task, which can influence inferences about optimality (Evans et al., 2019; Simen et al., 2009) – while using the same experimental task as (Evans & Brown, 2017).

## Experiment 1

### Method

It should be noted that the data for the “None-Time” and “None-Trial” groups were previously used in Crüwell and Evans (2021), where these groups were compared as part of an example for the preregistration template developed in that article.

#### Participants

For Experiment 1, 133 undergraduate students were recruited from the University of Newcastle, who completed the experiment online for course credit under protocols approved by the University of Newcastle Human Research Ethics Committee. Participants were assigned to one of four groups: “None-Time” (33), “None-Trial” (30), “RR-Time” (35), “RR-Trial” (35). We defined an exclusion criterion of 60% accuracy, where the 31 participants who scored below this criterion were excluded. Furthermore, we excluded only one further participant across the “None-Time” and “RR-Time” groups who completed less than 200 total trials across the entire task, as this would mean that they completed less than ten trials on average per block, making reliable block-by-block analyses of threshold (*a*) changes difficult. These exclusions resulted in 101 participants remaining for analysis (None-Time=27, None-Trial=23, RR-Time=26, RR Trial=25).

#### Task and procedure

The study was administered with either a fixed amount of time per block (“Time”) or a fixed number of trials per block (“Trial”), with participants either receiving explicit instructions that encourage them to pursue RR optimality (“RR”) or typical instructions from the decision-making literature with no clear performance goal (“None”); see Table 1 for an overview. For the “Time” groups, each block was 60 s in duration with participants completing as many trials as possible, and for the "Trial" groups, each block consisted of 40 trials for participants to complete at their own pace. Participants completed a total of 20 blocks, meaning that those in the “Trial” groups completed 800 trials, and those in the “Time” groups spent 1200 s (20 min) completing trials.

At commencement, participants were provided with general information about the study, then indicated their consent by continuing on to the task. Participants were then presented with a screen with general information about the task; a two-alternative forced-choice (2AFC) random dot kinematogram, where participants had to judge whether a cloud of dots appeared to be moving towards the top left or top right of the screen. After these initial instructions, those in the “None” groups then began the task, whilst those in the “RR” groups were presented with additional instructions explaining that they would receive points for correct answers, and encouraging them to get as many points as possible per minute^2^. During each trial, participants were presented with 40 white dots contrasted on a black background, where four dots (i.e., 10%) were moving coherently towards either the top left or top right of the screen, with dot movement and direction generated using the white-noise algorithm (Pilly & Seitz, 2009). Participants were told to press either the "z" key if they decided that the cloud of dots appeared to be moving to the top left, or the "/" key if they decided that the cloud of dots appeared to be moving to the top right.

Anticipatory responses, defined as responses quicker than 250 ms, produced a “Too Fast!” message, and the task timed out for 1500 ms before proceeding to the next trial. Depending on response accuracy, post-trial feedback produced either a “Correct” message presented for 500 ms, or an “Incorrect” message presented for 1500 ms, followed by a 500-ms inter-trial interval, before proceeding to the next trial. In Experiment 1, participants were given a “medium” (i.e., as defined in Evans & Brown, 2017) level of feedback after each block – from the end of block 4 onwards – summarising their RR performance.

#### Design and data analysis

We treated Experiment 1 as a 2 (Task Type: Time, Trial) * 2 (Instruction Type: RR, None) between-subjects design. We excluded responses faster than 250 ms and slower than 10,000 ms, assuming that these reflected anticipatory responses and lapses in attention, respectively. Some basic descriptive statistics for all three experiments can be seen in Table 2.

We used the full^3^ diffusion model (Ratcliff & Rouder, 1998; Ratcliff & Tuerlinckx, 2002) to estimate parameters for drift rate (*v*), threshold (*a*), starting point (*z*), and non-decision time (*ter*) – as well as between-trial variability in drift rate (*sv*), starting point (*sz*), and non-decision time (*ster*) – with two different model parameterisations used for qualitative and quantitative evaluation of data. The full diffusion model can account for characteristics commonly found in data from perceptual decision-making tasks, such as slow errors (e.g., Ratcliff & McKoon, 2008). Each of the four groups was assessed separately in their qualitative agreement with RR optimality, and then quantitative pairwise comparisons were used to determine whether some groups were closer to optimality than others.

**Table 2:** Descriptive statistics for Experiments 1, 2, 3

Group	MRT	PC	s/40trials	trials/60 s	RR
Exp1					
None-Trial	0.760	0.794	89.928	26.688	0.404
None-Time	0.880	0.720	102.261	23.469	0.333
RR-Trial	0.780	0.788	94.113	25.501	0.396
RR-Time	0.817	0.774	92.914	25.830	0.379
Exp2					
None-Trial	0.879	0.826	96.092	24.976	0.402
None-Time	0.814	0.832	93.519	25.663	0.420
RR-Trial	0.816	0.832	91.929	26.107	0.419
RR-Time	0.857	0.845	93.014	25.803	0.420
Exp3					
None-Trial	0.820	0.823	85.148	28.186	0.452
RR-Time	0.821	0.809	81.896	29.305	0.444

MRT - median reaction time; PC - proportion correct; "RR" in the group column refers to instruction type which encourages RR optimality; RR column provides numerical values for RR as per formula detailed in the main text.

RR was calculated as the accuracy rate divided by the average time spent on each trial; specifically, $\frac{PC}{MRT + ITI + FDT + (1-PC)*ET}$, where *MRT* reflects the mean response time, and *PC* reflects the accuracy, *ITI* reflects the inter-trial interval, *FDT* reflects the feedback display time, and *ET* reflects the additional timeout for error responses^4^. Optimal thresholds (*a*) were those that maximised RR across the range of 0.01 to 4 – in increments of 0.01 – and were determined as a function of the other parameters (*v*, *z*, *ter*, *sv*, *sz*, *ster*). As no solutions exist to determine the RR for each possible threshold in the full diffusion model (though see Bogacz et al., 2006 for a solution for the simple diffusion model), we determined the RR for each possible threshold through simulation using the method and framework of Evans (2019). Specifically, for the RR calculation of each possible threshold, we simulated 2000 trials with a step size of 10 ms and a maximum decision time of 10 s, which created an extremely large computational burden in our quantitative analysis.

In all cases, models were estimated through Bayesian hierarchical model estimation (Shiffrin et al., 2008), where the parameter estimates of each individual are constrained to follow group-level distributions. The exact model structures and prior specifications can be seen in the Supplementary Materials. The posterior distributions were estimated through differential-evolution Markov chain Monte Carlo (DE-MCMC; Ter Braak, 2006; Turner et al., 2013), which allows for efficient movement through correlated dimensions by using the current samples from each chain to inform the proposals of other chains. The estimation process used 3*k* chains, where *k* is the number of free parameters per individual subject, and sampled for a total of 4000 iterations, with the first 1000 iterations discarded as burn-in^5^. In all cases, the focus of estimation and inference was on the group-level parameters relating to threshold: the estimated threshold (*a*), and/or the distance of the estimated threshold from the optimal threshold.

The qualitative assessment involved estimating a model where threshold (*a*) was allowed to vary across blocks, with these block-by-block group-level posterior estimates of threshold compared to the group-level distribution of the optimal threshold^6^. The group-level distribution of the optimal threshold was calculated by taking all of the group-level posterior samples for *v*, *z*, and *ter*, and calculating the respective optimal *a* value for each sample, which formed an optimal threshold “posterior” distribution. The estimated posterior distributions for thresholds for each block, and the calculated “posterior” distributions for the optimal threshold across all blocks, were plotted for each group to qualitatively assess whether their thresholds get closer to optimality over time, how their thresholds differ from optimality (e.g., generally more cautious, optimal, or overly urgent), and whether the groups seem to differ in their proximity to optimality.

To ensure that the differences between actual and optimal thresholds did not differ too greatly between the group level and individual level, we also performed the qualitative analyses for each experiment at the individual level, which provided qualitatively matching results to the equivalent group-level analyses (see the Supplementary Materials).

The quantitative assessment was only performed on the data from blocks 11–20, assuming that participants had approximately converged on their chosen threshold by block 11, though perhaps not beforehand, based on the plots from the qualitative analysis. This involved estimating the difference from optimality for each participant (i.e., $c_{\text {i}}$ = $a_{\text {i}}$ - $b_{\text {i}}$, where $b_{\text {i}}$ is the optimal threshold for person *i*), and a group-level distribution for the difference parameter *c*. To make inferences about which groups appeared to be closer to optimality than other groups, we estimated models for the pairwise comparisons between the four different groups, with $\Delta _c$ providing the estimate of the difference from optimality between the two groups. To provide inferences, we used the Savage–Dickey density ratio method (Wagenmakers et al., 2010) to approximate the Bayes factors.

In total, we estimated 18 different models for Experiments 1 and 2: specifically, for each of the six pairwise comparisons between groups, we estimated three different types of models, aimed at helping to provide three different types of inferences. The three models comprised: (1) a model with a normally distributed prior that was used for parameter estimation of the $\Delta _c$ parameter, and provided inferences about whether or not an effect appeared to be present; (2) a model that restricted the prior of $\Delta _c$ in one direction (i.e., normally distributed prior with positive truncation), providing a model comparison between the hypotheses of group A being closer to optimality than group B, or there being no effect (“A vs Null”); (3) a model that restricted the prior of $\Delta _c$ in the other direction (normally distributed prior with negative truncation), providing a model comparison between the hypotheses of group B being closer to optimality than group A, or there being no effect (“B vs Null”). As the model with a normally distributed prior can only tell us if an effect is present, the two directional models (with truncated priors) can provide further insight into which of the two groups the effect is most likely to favour.

Furthermore, as the Bayes factor is transitive, we also calculated an “A vs B” Bayes factor based on the previous two Bayes factors (i.e., $\frac{\text{ A } \text{ vs } \text{ Null }}{\text{ B } \text{ vs } \text{ Null }}$ ), comparing hypothesis A (i.e., group A is closer to optimality than group B) against hypothesis B (i.e., group B is closer to optimality than group A), to provide a direct assessment of whether fixed-time or fixed-trial appears most likely to be superior.^7^

The strength of evidence provided by the Bayes factors was interpreted following van Doorn et al. (2020) and Jeffreys (1961). Bayes factors less than 3 or greater than $\frac{1}{3}$ in magnitude indicate weak evidence, between 3 and 10 or $\frac{1}{3}$ - $\frac{1}{10}$ indicate moderate evidence, between 10 and 100 or $\frac{1}{10}$ - $\frac{1}{100}$ indicate strong evidence, and greater than 100 or less than $\frac{1}{100}$ indicate decisive evidence. Bayes factors above 1 indicate evidence for the alternate hypothesis, and Bayes factors below 1 indicate evidence for the null hypothesis.

### Results

**Fig. 1:**
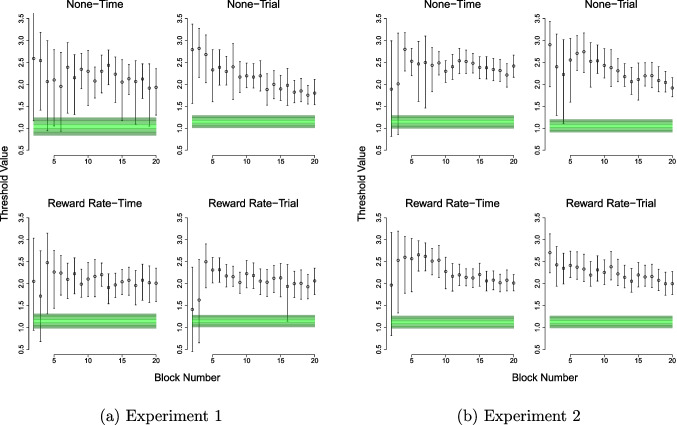
Actual threshold values for each condition, compared to their optimal threshold. *Circles* represent median group-level posterior threshold for each block (starting at block 2) with *error bars *showing the 95% quantiles. This is compared with the group-averaged optimal threshold indicated by the coloured bands delineated by different *shades of green*. The *lightest, centre band* represents the 40–60% quantile, the *middle shade represents* the 20–40% and 60–80% quantiles, and the *darkest shade* the 10–20% and 80–90% quantiles. Thresholds above the optimal band indicate cautious behaviour, whilst those below the band indicate more urgent behaviour

For the qualitative assessment, plots comparing the actual threshold to optimal threshold for each group revealed two general trends: that all groups were more cautious than optimality, and that all groups generally came closer to optimality over the later blocks (see Fig. 1a). The None-Trial group most closely exhibited these trends, and also came the closest to achieving optimality, though the posterior median for their actual thresholds always remained outside of the bands of their optimal distribution. In contrast, trends for the None-Time group appeared to be the most variable and the furthest from achieving optimality, with trends for the two RR groups somewhere between the two None groups. The first few blocks of each group also showed higher variability in median threshold values, with some point estimates nearly at optimal, likely reflecting some initial threshold adjustments while participants adapted to the task.

**Fig. 2:**
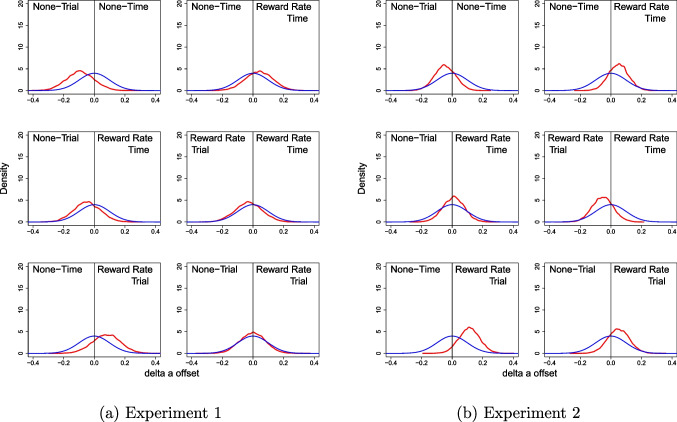
Prior-posterior distribution density plots of the group-level $\Delta _c$ parameter using the normal prior distribution. The prior distributions are in *blue*, and the posterior distributions are in *red*

For the quantitative assessment, Bayes factors based on the Savage–Dickey ratios for the pairwise comparisons between groups are presented in Table 3, with graphical representation of the estimated posterior distributions for the model with a normally distributed prior in Fig. 2a. When only looking at the comparisons of any effect against the null, all comparisons showed weak evidence for either an effect or the null. However, when looking at directional hypotheses, we found moderate evidence in favour of None-Trial being closer to optimality than None-Time (compared to the null and the inverse), and moderate evidence in favour of RR-Trial being closer to optimality than None-Time (compared to the null and the inverse). Importantly, these results seem to suggest that people are *at least* as close to optimality under fixed-trial conditions as they are under fixed-time conditions, and people are potentially even closer to optimality under fixed-trial conditions compared to fixed-time conditions – though the latter inference could be due to the poorer performance of the None-Time group.

## Experiment 2

### Method

All methodological details of Experiment 2 were identical to Experiment 1, apart from the deviations noted here. Most notably, in Experiment 2 participants were given a “low” level of feedback (i.e., as defined in Evans & Brown, 2017) after each block, where they were not given any information about their performance^8^. For Experiment 2, 230 undergraduate students were recruited from the University of Queensland, who completed the experiment online for course credit under protocols approved by the University of Queensland Human Research Ethics Committee. Participants were assigned to one of four groups: “None-Time” (66), “None-Trial” (49), “RR-Time” (55), “RR-Trial” (60). We again defined an exclusion criterion of 60% accuracy, where the 47 participants who scored below this criterion were excluded. Furthermore, we excluded only one further participant across the “None-Time” and “RR-Time” groups who completed less than 200 total trials across the entire task, as this would mean that they completed less than ten trials on average per block, making reliable block-by-block analyses of threshold (*a*) changes difficult. These exclusions resulted in 182 participants remaining for analysis (None-Time=50, None-Trial=41, RR-Time=43, RR Trial=48).

**Table 3:** Savage–Dickey ratios for Experiment 1, comparing how groups differ in their distance from optimality

Prior distribution							
Group combination	Normal	Positive truncation		Negative truncation		Ratio	
(A/B)	Effect vs Null	A vs Null	A vs Null (adj)	B vs Null	B vs Null (adj)	A vs B	A vs B (adj)
None-Time/**None-Trial**	1.564	1.005	0.505	4.388	4.276	0.229	0.118
**RR-Time**/None-Time	1.016	2.701	1.253	1.378	1.339	1.960	0.936
RR-Time/**None-Trial** cf. SR2012	1.104	1.272	0.651	2.812	2.724	0.452	0.239
RR-Time/**RR-Trial** cf. EB2017	0.952	1.226	0.674	2.554	2.517	0.480	0.268
**RR-Trial**/None-Time	1.385	4.300	1.830	1.159	1.123	3.709	1.629
RR-Trial/**None-Trial**	0.818	1.520	0.810	1.652	1.601	0.920	0.506

**Bold** indicates which of the two groups is more likely to produce an optimal decision strategy; SR2012 - Starns & Ratcliff, 2012; EB2017 - Evans & Brown, 2017; Normal Distribution = effect vs. no effect; Positive truncation (directional) = effect in favour of group A vs. no effect; Negative truncation (directional) = effect in favour of group B vs. no effect; ratio = effect in favour of group A vs. effect in favour of group B; adj = adjusted Bayes factors to compensate for densities very close to zero which may be higher than at zero; RR = Reward Rate

### Results

For the qualitative assessment, plots comparing actual threshold to optimal threshold for each group revealed the same two general trends as Experiment 1: that all groups were more cautious than optimality (even more so than Experiment 1), and that all groups became closer to optimality over blocks (see Fig. 1b). In contrast to Experiment 1, the RR-Trial group appeared to most closely resemble these trends, followed by the RR-Time and None-Trial groups. The first few blocks of each group also showed higher variability in median threshold values, similar to Experiment 1.

For the quantitative assessment, Bayes factors based on the Savage–Dickey ratios for the pairwise comparisons between groups are presented in Table 4, with graphical representation of the estimated posterior distributions for the model with a normally distributed prior in Fig. 2b. When only looking at the comparisons of any effect against the null, all comparisons showed weak evidence for the null, except for the RR-Trial /None-Time group, which showed weak evidence for an effect.

However, when looking at directional hypotheses, we found strong evidence in favour of RR-Trial being closer to optimality than None-Time (compared to the null and the inverse), and moderate evidence in favour of RR-Trial being closer to optimality than None-Trial (compared to the null and the inverse) and RR-Time (compared to the inverse), and moderate evidence in favour of RR-Time being closer to optimality than None-Time (compared to the inverse). Importantly, these results again seem to suggest that people are *at least* as close to optimality under fixed-trial conditions as they are under fixed-time conditions, and people are potentially even closer to optimality under fixed-trial conditions compared to fixed-time conditions.

**Table 4:** Savage–Dickey ratios for Experiment 2, comparing how groups differ in their distance from optimality

Prior distribution							
Group combination	Normal	Positive truncation		Negative truncation		Ratio	
(A/B)	Effect vs Null	A vs Null	A vs Null (adj)	B vs Null	B vs Null (adj)	A vs B	A vs B (adj)
None-Time/**None-Trial**	0.898	0.834	0.462	2.676	2.637	0.311	0.175
**RR-Time**/None-Time	0.860	2.768	1.190	0.797	0.785	3.473	1.517
RR-Time/None-Trial cf. SR2012	0.709	1.444	0.704	1.401	1.361	1.031	0.517
RR-Time/**RR-Trial** cf. EB2017	0.940	0.780	0.415	2.845	2.765	0.274	0.150
**RR-Trial**/None-Time	2.612	12.690	4.047	0.556	0.539	22.835	7.515
**RR-Trial**/None-Trial	0.904	3.196	1.324	0.933	0.906	3.427	1.462

**Bold** indicates which of the two groups is more likely to produce an optimal decision strategy;  SR2012 - Starns & Ratcliff, 2012; EB2017 - Evans & Brown, 2017; Normal distribution = effect vs. no effect; Positive truncation (directional) = effect in favour of group A vs. no effect; Negative truncation (directional) = effect in favour of group B vs. no effect; ratio = effect in favour of group A vs. effect in favour of group B; adj = adjusted Bayes factors to compensate for densities very close to zero which may be higher than at zero; RR = Reward Rate

**Fig. 3:**
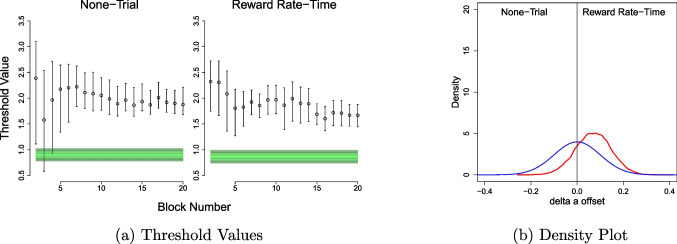
Experiment 3 comparisons of RR-Time/ None-Trial group combination. **a** Actual threshold values for each condition, compared to their optimal threshold. **b** Prior-posterior distribution density plot of the group-level $\Delta _c$ parameter using the normal prior distribution

## Experiment 3

Interestingly, the results of both Experiments 1 and 2 indicate that people are *at least* as close to optimality under fixed-trial conditions as they are under fixed-time conditions; that is, that people are either closer to optimality under fixed-trial conditions or have equivalent proximity to optimality under both conditions. Importantly, these results suggest that people are *not* able to better optimise RR under fixed-time conditions. Experiment 3 attempts to address two key remaining differences between our study and the study of Starns and Ratcliff (2012). Specifically, while our previous experiments both provided manipulations of whether the instructions gave participants the goal of RR optimality, our RR emphasising instructions did differ from those of Starns and Ratcliff (2012); for example, Starns and Ratcliff (2012) explicitly instructed participants to ignore their errors, whereas our previous instructions only explained to participants what they should optimise. Furthermore, our previous experiments included a fairly large timeout for incorrect responses, where error feedback was presented for 1000 ms longer than correct feedback (1500 ms vs 500 ms), which pushes the optimal strategy towards being more cautious and avoiding errors, whereas Starns and Ratcliff (2012) presented correct and error feedback for an equal amount of time (300 ms each, with an inter-trial interval of 100 ms). In Experiment 3, we address these differences by using instructions for the fixed-time group that are directly based on those of Starns and Ratcliff (2012), and removing the error timeout from our previous experiments. However, it should be noted that there are still several differences between our Experiment 3 and Starns and Ratcliff (2012). For example, Starns and Ratcliff (2012) also included a between-block manipulation of difficulty – allowing them to have a more nuanced assessment of optimality regarding whether participants adapted to the difficult conditions in an optimal manner – as well as an assessment of aging effects, and utilised a different experimental task.

### Method

All methodological details of Experiment 3 were identical to Experiment 1, apart from the deviations noted here. Most notably, we simplified our design to only contain two groups that focused on the key comparison of interest: a RR-Time group, who received RR instructions based on those from Starns and Ratcliff (2012) (see the Supplementary Materials for the exact instructions), and a None-Trial group, which were given the same instructions as the None-Trial groups from the previous experiments. Furthermore, post-trial feedback for both correct and incorrect responses was presented for 500 ms. For Experiment 3, 71 undergraduate students were recruited from the University of Queensland, who completed the experiment online for course credit under protocols approved by the University of Queensland Human Research Ethics Committee. Participants were assigned to one of two groups: “None-Trial” (35) or “RR-Time” (36). We again defined an exclusion criterion of 60% accuracy, where the 13 participants who scored below this criterion were excluded. These exclusions resulted in 58 participants remaining for analysis (None-Trial=28, RR-Time=30).

### Results

For the qualitative assessment, plots comparing actual threshold to optimal threshold for each group revealed the same two general trends as Experiments 1 and 2 (see Fig. 3a). First, both groups were more cautious than optimality, and to a greater extent than Experiments 1 and 2 as the optimal band in Experiment 3 reflected an even more urgent strategy. Second, both groups became closer to optimality over blocks, though in contrast to Experiments 1 and 2, the fixed-time group – in this case, RR-Time – appeared to come closest to optimality. For the quantitative assessment, the results showed weak evidence for an effect between groups (Bayes factor [Effect vs Null] = 1.14; see also Fig. 3b), with the direct comparison between hypotheses that posited one group was closer to optimality than the other showing moderate evidence in favour of RR-Time being closer to optimality than None-Trial (Bayes factor [A vs B] = 4.24). Furthermore, it should be noted that the results of the simple diffusion model came to somewhat different conclusions, showing weak evidence for no differences between groups in all cases (see the Supplementary Materials), though our key interpretations focus on the full diffusion model. These results contrast with those of Experiment 1 and 2, and while they do not show a clear superiority of the fixed-time condition, they do indicate that people are *not* at least as optimal in the fixed-trial condition, suggesting that task instructions and/or pace may influence the conclusions.

## Discussion

The current study aimed to provide a more comprehensive investigation into whether people behave more optimally in fixed-time designs compared to fixed-trial designs. While previous studies including both of these designs have been limited and showed mixed results (Starns & Ratcliff, 2012; Evans & Brown, 2017), several other studies have exclusively used fixed-time conditions (Balci et al., 2011; Bogacz et al., 2010; Simen et al., 2009), showcasing an assumption in the literature that fixed-time designs are better suited than fixed-trial designs to studying RR optimality. The findings of Experiments 1 and 2 indicate that this is not the case, providing evidence that people were *at least* as close to optimality under fixed-trial conditions as they were under fixed-time conditions. However, the findings of Experiment 3 contrast with those in Experiments 1 and 2, providing evidence that people were *not* at least as close to optimality under fixed-trial conditions as they were under fixed-time conditions. Importantly, Experiment 3 utilised task instructions and pacing that better matched those of Starns and Ratcliff (2012); fixed-time participants were explicitly instructed to ignore errors, but fixed-trial participants were not given any specific instructions on how to perform the task. Together, these findings indicate that people are generally *at least* as good at optimizing RR under fixed-trial compared to fixed-time conditions, but that specific differences in instructions between groups may cause a superiority of fixed-time conditions. Furthermore, these findings may also be reflective of a general suggestion made by Evans et al. (2019); that people may actually be fairly resistant to changing their level of caution in many situations that can influence optimality, with fixed-time/fixed-trial designs potentially being one of these situations.

Our findings also provide further evidence for a common conclusion in the literature: that participants are generally more cautious than RR optimality dictates that they should be (Evans & Brown, 2017; Balci et al., 2011; Starns & Ratcliff, 2012, 2010). Across all three experiments, we found that all groups were sub-optimally cautious, which became even more extreme in Experiment 3 where the error timeout was removed and an even more urgent strategy was required for optimality.

Importantly, the latter finding reiterates the conclusion of some previous studies that have suggested that inference about participants’ ability to achieve optimality may be dependent on task design, and more specifically, that the timeouts in the task can greatly influence how close people come to achieving RR optimality (Evans et al., 2019; Simen et al., 2009). Furthermore, while participants also generally moved closer to optimality over time – another common finding in the literature (Evans & Brown, 2017; Evans et al., 2019)– it is unclear how much of the change over time is due to an increased understanding of how to achieve optimality, or a range of other factors that would also cause a decrease in threshold, such as fatigue and/or boredom (Agrawal et al., 2022).

More broadly, these findings further showcase the importance of better understanding *why* participants are sub-optimally cautious. While the current study seems to generally indicate that a specific factor – fixed time vs fixed trial designs – does not influence conclusions about optimality, our knowledge remains limited on the broader questions of why people are sub-optimal, and whether they can adapt to become optimal. While some previous studies have found people to behave approximately optimal in some conditions (Evans et al., 2019; Simen et al., 2009), these were cases where the task design was modified to make the optimal strategy a more cautious one, meaning that participants may have only been close to optimality by default, rather than by choice. Based on the findings of Evans and Brown (2017), where people came close to optimality when given specific feedback on how to adapt their performance, it is possible that participants are simply unaware of how exactly to optimise their performance. However, even with this extreme level of guidance people were still slightly too cautious, suggesting that making people aware of the optimal strategy may not be completely sufficient. Another potential explanation is that participants are simply not motivated to achieve optimality, and simply desire to get out of the experiment as quickly as possible (e.g., the Min-RT strategy; Hawkins et al., 2012). However, this explanation seems inconsistent with the general finding of people being sub-optimally cautious, as under this explanation, we would expect people to be sub-optimally *urgent*. While monetary rewards may better motivate individuals to achieve reward rate optimality rather than relying on their default strategy, non-monetary reward-based instructions are common in other decision-making tasks and typically yield high compliance rates, as seen in instruction-based manipulations of the speed-accuracy trade-off (e.g., Evans, 2021), suggesting that participants do not *need* monetary incentives to comply with instructions when they understand how to comply with them. More generally, while findings seem to indicate that RR optimality cannot provide a general explanation for how people behave, we believe that understanding why people are sub-optimal is an important research direction for understanding human behaviour.

## Open Practices Statement

The data and code for each experiment are available at https://osf.io/8s6pn/, and none of the experiments were preregistered.

## Supplementary Information

Below is the link to the electronic supplementary material.

Supplementary file 1 (pdf 530 KB)

## Data Availability

The data for all experiments are available on https://osf.io/8s6pn/

## Footnotes

1 Note that this explanation does not account for the qualitative disagreements with RR optimal behaviour in some circumstance, such as the findings of Starns and Ratcliff (2012), where participants were more cautious in more difficult blocks, when RR optimality dictates that they should be less cautious.

2 Instruction transcripts for all experiments can be found in the Supplementary Materials

3 Parameters were also estimated using the simple diffusion model (Ratcliff, 1978), with results provided in the Supplementary Materials. In general, the full and simple diffusion models come to the same qualitative conclusions in Experiments 1 and 2, though differed slightly in Experiment 3. Specifically, in Experiment 3 the simple diffusion model showed no evidence for any superiority of either design, whereas the full diffusion model showed moderate evidence that fixed-trial design was not at least as good as the fixed-time design.

4 Note that we also performed an analysis on RR to see whether it differed between groups, which can be seen in the Supplementary Materials. While there was moderate evidence for RR being higher in fixed-trial compared to fixed-time conditions in Experiment 1, all other comparisons in all three experiments showed evidence against a difference in RR between groups.

5 Note that we also used a migration algorithm to help prevent stuck chains, which occurred every 25 iterations between iterations 300 and 800.

6 Note that while drift rate could technically also vary across blocks, which would change the optimal threshold distribution for each block, (1) this greatly increases the complexity of the model making it more computationally taxing and difficult to estimate, and (2) the results of Evans and Brown (2017) suggested that allowing drift rate to vary does not influence the results, as the optimal distribution does not systematically change over blocks

7 Also note that in addition to the regular Savage–Dickey ratio, which only contrasts the densities of the posterior and the prior at 0, we also calculated a more conservative (i.e., more favourable to the null hypothesis) “adjusted” version, rather than calculating the Bayes factor based on the posterior density at exactly 0, instead uses the highest posterior density within a band very close to 0 (i.e., in our case, -0.02 < x < 0.02). While this calculation is less theoretically correct – and so we only present it alongside the regular Savage–Dickey ratio, and base our interpretations on the regular Savage–Dickey ratio – it provides some additional robustness against situations where the posterior density is quite peaked near 0, but not exactly at 0.

8 Though note that participants were still given trial-by-trial feedback.

## Code availability

R code for the study is available on https://osf.io/8s6pn/
