# Supplementary material for: People are at least as good at optimizing reward rate under equivalent fixed-trial compared to fixed-time conditions
Source: Psychon Bull Rev. 2025 Apr 3;32(5):2124–35. doi: 10.3758/s13423-025-02680-y (PMC12426136; doi:10.3758/s13423-025-02680-y)
Supplement: Supplementary file 1 — (pdf 530 KB) [file 13423_2025_2680_MOESM1_ESM.pdf]

Supplementary Materials for: People are at least as good at optimizing reward rate under equivalent fixed-trial compared to fixed-time conditions

Grant J. Taylor[a], Scott D. Brown[b], and Nathan J. Evans[abc]

[a] School of Psychology, University of Queensland, Australia

[b] School of Psychology, University of Newcastle, Australia

[c] Department of Psychology, University of Liverpool, UK

Hierarchical Model Structure

The model used for qualitative assessment of each of the four groups (None-Time, None-Trial, Reward Rate-Time, Reward Rate-Trial) had the following structure and priors:

$$
\begin{aligned}
&\text{Data level:} && \\
(RT_i, resp_i) &\sim \text{Diffusion}(v_i, z_i, ter_i, a_{j,i}) \\
&\text{Group level:} && \\
v_i &\sim N(\mu_v, \sigma_v) \\
\frac{z_i}{a_{j,i}} &\sim TN(\mu_{\frac{z}{a}}, \sigma_{\frac{z}{a}}, 0, 1) \\
ter_i &\sim TN(\mu_{ter}, \sigma_{ter}, 0, \infty) \\
a_{j,i} &\sim TN(\mu_{a_j}, \sigma_{a_j}, 0, \infty) \\
&\text{Prior Distributions:} && \\
\mu_v &\sim N(3, 1) \\
\mu_{\frac{z}{a}} &\sim TN(0.5, 0.1, 0, 1) \\
\mu_{ter} &\sim TN(0.3, 0.1, 0, \infty) \\
\mu_{a_j} &\sim TN(1.5, 0.5, 0, \infty) \\
\sigma_v, \sigma_{\frac{z}{a}}, \sigma_{ter}, \sigma_{a_j}, &\sim \Gamma(1, 1)
\end{aligned}
$$

where subscript i indexes the participants, subscript j indexes blocks (2 to 20), N is the normal distribution with parameters (mean, standard deviation), TN is the truncated normal distribution with parameters (mean, standard deviation, lower bound, upper bound), and Γ is the gamma distribution with parameters (shape, scale).

The model used for quantitative assessment of the six group comparisons (Reward Rate-Trial/None-Trial, Reward Rate-Trial/None-Time, Reward Rate-Time/Reward Rate-Trial, Reward Rate-Time/None-Trial, Reward Rate-Time/None-Time, None-Time/None-Trial) had the following structure and priors:

$$
\begin{aligned}
&\text{Data level:} && \\
(RT_i, resp_i) &\sim \text{Diffusion}(v_i, a_i, ter_i, z_i) \\
&\text{Group level (Group A):} && \\
v_i &\sim N(\mu_v, \sigma_v) \\
\frac{z_i}{a_i} &\sim TN(\mu_{\frac{z}{a}}, \sigma_{\frac{z}{a}}, 0, 1) \\
ter_i &\sim TN(\mu_{ter}, \sigma_{ter}, 0, \infty) \\
c_i &\sim N(\mu_c - \Delta_c, \sigma_c) \\
&\text{Group level (Group B):} && \\
v_i &\sim N(\mu_v, \sigma_v) \\
\frac{z_i}{a_i} &\sim TN(\mu_z, \sigma_z, 0, 1) \\
ter_i &\sim TN(\mu_{ter}, \sigma_{ter}, 0, \infty) \\
c_i &\sim N(\mu_c + \Delta_c, \sigma_c) \\
&\text{Prior Distributions:} && \\
\mu_v &\sim N(3, 1) \\
\mu_{\frac{z}{a}} &\sim TN(0.5, 0.1, 0, 1) \\
\mu_{ter} &\sim TN(0.3, 0.1, 0, \infty) \\
\mu_c &\sim N(0, 0.2) \\
\mu_{\Delta_c} &\sim N(0, 0.1) \\
\text{(A vs Null): } \mu_{\Delta_c} &\sim TN(0, 0.1, 0, \infty) \\
\text{(B vs Null): } \mu_{\Delta_c} &\sim TN(0, 0.1, -\infty, 0) \\
\sigma_{\frac{z}{a}}, \sigma_{ter}, \sigma_v, \sigma_a, \sigma_{\Delta_c} &\sim \Gamma(1, 1)
\end{aligned}
$$

where Δ_c is a group-level parameter that reflects the difference between the two groups in the c parameter (i.e., the difference between the two groups in their difference from optimality), and subscript i indexes the participants. Given that the qualitative analysis indicated that all groups were sub-optimally too cautious, this meant that positive Δ_c values indicated that Group A was closer to optimality than Group B, whereas negative Δ_c values indicated that Group B was closer to optimality than Group A, resulting in the prior definitions for the "A vs Null" and "B vs Null" directional tests (i.e., if all group were sub-optimally too urgent, then the prior distributions for these tests would be reversed, and if different groups were sub-optimal in different directions, then these directional tests would not provide clear inferences regarding relative proximity to optimality).

Instructions and feedback scripts

Experiment 1

Fixed time condition

Welcome to the experiment! In this experiment, we examine your ability to make fast and accurate decisions about some moving dots. At each trial, a cloud of dots will appear in the centre of the screen. Your job is to determine whether the dots are moving toward the top left or top right of screen. To select 'left' press the 'Z' key, and to select 'right' press the ' / ' (i.e., question mark) key. Additionally, throughout the experiment you will receive feedback onhow you are currently performing. There are " + nBlocks + " blocks, and each block will go for " + blockTime/60000 + " minutes. Therefore, the total number of trials completed will depend on the amount of time that you take on each trial.

Fixed trial condition

Welcome to the experiment!" In this experiment, we examine your ability to make fast and accurate decisions about some moving dots. At each trial, a cloud of dots will appear in the centre of the screen. Your job is to determine whether the dots are moving toward the top left or top right of screen. To select 'left' press the 'Z' key, and to select 'right' press the ' / ' (i.e., question mark) key. Additionally, throughout the experiment you will receive feedback on how you are currently performing. There are " + nBlocks + " blocks of " + nTrials + " trials. Therefore, you will make a total of " + (nBlocks*nTrials) + " decisions.

Extra instructions (in red font) – only participants in groups with reward rate instructions

Your goal is to attain as many correct answers as quickly as you can. You will receive 1 point for each correct answer, and you should try to get as many points as you can in each minute period. Remember that being too cautious may take too long and take away opportunities for points in each minute. Also, being too quick may cause you to be less accurate and take away total points.

Block feedback (from block 5 onwards) – all participants

Well done! You're " + (nCurrentBlock-1) + " of " + nBlocks + " blocks through the experiment! In recent blocks you've attained " + nCorrectResponses + " points in " + experimentMins + " minutes, meaning you achieved " + pointsPerMin + " points per minute.

Experiment 2

Fixed time condition

Welcome to the experiment! In this experiment, we examine your ability to make fast and accurate decisions about some moving dots. At each trial, a cloud of dots will appear in the centre of the screen. Your job is to determine whether the dots are moving toward the top left or top right of screen. To select 'left' press the 'Z' key, and to select 'right' press the ' / ' (i.e., question mark) key. Additionally, throughout the experiment you will receive feedback onhow you are currently performing. There are " + nBlocks + " blocks, and each block will go for " + blockTime/60000 + " minutes. Therefore, the total number of trials completed will depend on the amount of time that you take on each trial.

Fixed trial condition

Welcome to the experiment!" In this experiment, we examine your ability to make fast and accurate decisions about some moving dots. At each trial, a cloud of dots will appear in the centre of the screen. Your job is to determine whether the dots are moving toward the top left or top right of screen. To select 'left' press the 'Z' key, and to select 'right' press the ' / ' (i.e., question mark) key. Additionally, throughout the experiment you will receive feedback on how you are currently performing. There are " + nBlocks + " blocks of " + nTrials + " trials. Therefore, you will make a total of " + (nBlocks*nTrials) + " decisions.

Extra instructions (in red font) – only participants in groups with reward rate instructions

Your goal is to attain as many correct answers as quickly as you can. You will receive 1 point for each correct answer, and you should try to get as many points as you can in each minute period. Remember that being too cautious may take too long and take away opportunities for points in each minute. Also, being too quick may cause you to be less accurate and take away total points.

Block feedback

Block feedback omitted.

Experiment 3

Fixed time condition

Welcome to the experiment! In this experiment, we examine your ability to make fast and accurate decisions about some moving dots. At each trial, a cloud of dots will appear in the centre of the screen. Your job is to determine whether the dots are moving toward the top left or top right of screen. To select 'left' press the 'Z' key, and to select 'right' press the ' / ' (i.e., question mark) key. Additionally, throughout the experiment you will receive feedback onhow you are currently performing. There are " + nBlocks + " blocks, and each block will go for " + blockTime/60000 + " minutes. Therefore, the total number of trials completed will depend on the amount of time that you take on each trial.

Fixed trial condition

Welcome to the experiment!" In this experiment, we examine your ability to make fast and accurate decisions about some moving dots. At each trial, a cloud of dots will appear in the centre of the screen. Your job is to determine whether the dots are moving toward the top left or top right of screen. To select 'left' press the 'Z' key, and to select 'right' press the ' / ' (i.e., question mark) key. Additionally, throughout the experiment you will receive feedback on how you are currently performing. There are " + nBlocks + " blocks of " + nTrials + " trials. Therefore, you will make a total of " + (nBlocks*nTrials) + " decisions.

Extra instructions (in green font) – fixed time condition only

Your goal is to attain as many correct answers as quickly as you can. We are not concerned with the number of errors that you make - we only count your correct responses. You will receive 1 point for each correct answer, and you should try to get as many points as you can in each minute period. To get the most points, you should try to be both fast and accurate in your responding. However, if you are overly slow, you will not complete many trials before time is up, and you will have fewer opportunities to make correct responses. So, going faster may help you get more correct responses even if it also leads to more errors.

Block feedback (from block 5 onwards) – fixed time and fixed trial conditions

Well done! You're " + (nCurrentBlock-1) + " of " + nBlocks + " blocks through the experiment! In recent blocks you've attained " + nCorrectResponses + " points in " + experimentMins + " minutes, meaning you achieved " + pointsPerMin + " points per minute.

Starns & Ratcliff (2012)

Extra instructions (from Starns & Ratcliff, 2012, pg.141) – fixed time condition only

Each block of trials will last for 30 seconds, and you will keep getting new trials until time is up. Try to get as many correct answers as possible within each block. We are not concerned with the number of errors that you make – we only count your correct responses. To get the most correct answers, you should try to be both fast and accurate in your responding. If you are overly slow, you will not complete many trials before time is up, and you will have fewer opportunities to make correct responses. So, going faster may

help you get more correct responses even if it also leads to more errors.

Analysis with Simple Diffusion Model

We initially favoured the simple diffusion model over the full diffusion model (e.g., Ratcliff, 1978; Ratcliff & Rouder, 1998) as the goal of the current study was Model Application (see Crüwell, Stefan, & Evans, 2019), and the simple diffusion model (1) has been shown to possess good measurement properties compared to its more complex counterparts (Lerche & Voss, 2016; van Ravenzwaaij, Donkin, & Vandekerckhove, 2017; Boehm et al., 2018; Evans, Tillman, & Wagenmakers, 2020), and (2) allows for the reward rate to be analytically solved for a given v, a, z, and ter (Bogacz, Brown, Moehlis, Holmes, & Cohen, 2006), which is necessary for our quantitative evaluation. Based on the suggestions of the reviewers and the editor, we undertook a subsequent analysis using the full diffusion model, which can provide a good account of data with trends such as slow errors, which are likely to arise in tasks such as the random dot motion task. While we base our conclusions on the results using the full diffusion model reported in the main text, we report the results of the simple diffusion model here.

Note that estimation of optimal thresholds for the qualitative plots using the full diffusion model were based on simulating n=5000 samples, compared to n=10000 for the simple diffusion model, as computation of the full diffusion model was too computationally onerous to go any higher. To ensure robustness, we also completed estimation of optimal threshold bands at n= 100, 200, and 1000, finding no difference in positioning of the threshold band, only an increase in variation as the n value decreased. From this we inferred analysis at n=5000 for the full diffusion model would be adequate to draw conclusions.

Experiment 1

Plots comparing actual threshold to optimal threshold for each group revealed two general trends: that all groups were more cautious than optimality, and that all groups

became closer to optimality over blocks (see Figure 1a). The None-Trial group appeared to come the closest to achieving optimality, though the posterior median for their actual thresholds always remained outside of the bands of their optimal distribution. In contrast, the None-Time group appeared to be the furthest from achieving optimality, with the two Reward Rate groups somewhere in between the two None groups. While the Reward Rate-Trial group appears to be very close to optimality in the first two blocks, the threshold estimates in these blocks appear to be outliers reflecting some initial threshold adjustments while participants adapted to the task, as afterwards the threshold estimates are consistently much further from optimality.

Bayes factors based on the Savage-Dickey Ratios for the pairwise comparisons between groups are presented in Table 1, with graphical representation of the estimated posterior distributions for the standard model with a normal prior in Figure 2a. When only looking at the comparisons of any effect against the null, all comparisons showed weak evidence for either an effect or the null, apart from the None-Time/None-Trial comparison showing moderate evidence for an effect, with the posterior estimations in Figure 2a suggesting that this was in favour of None-Trial being closer to optimality than None-Time. However, when looking at directional hypotheses (i.e., Group A/B is closer to optimality than Group B/A) compared to the null, we found strong evidence in favour of None-Trial being closer to optimality than None-Time (compared to the null), moderate evidence for both Reward Rate groups being closer to optimality than None-Time (compared to the null), and moderate evidence in favour of None-Trial being closer to optimality than Reward Rate-Time (compared to the null). Most notably, when looking at a direct comparison between hypotheses that posited one group was closer to optimality than the other, we again found strong evidence in favour of None-Trial being closer to optimality than None-Time (compared to the inverse), moderate evidence for both Reward Rate groups being closer to

optimality than None-Time (compared to their respective inverses), and moderate evidence in favour of None-Trial being closer to optimality than Reward Rate-Time (compared to the inverse).

Experiment 2

Plots comparing actual threshold to optimal threshold for each group revealed the same two general trends as Experiment 1: that all groups were more cautious than optimality (even more so than Experiment 1), and that all groups became closer to optimality over blocks (see Figure 1b). In contrast to Experiment 1, the Reward Rate-Trial group appeared to come closest to optimality, with the ordering of the other three groups more unclear.

Bayes factors based on the Savage-Dickey Ratios for the pairwise comparisons between groups are presented in Table 2, with graphical representation of the estimated posterior distributions for the standard model with a normal prior in Figure 2b. When only looking at the comparisons of any effect against the null, all comparisons showed weak evidence for the null, suggesting ambiguous evidence in all comparisons. However, when looking at directional hypotheses (i.e., Group A/B is closer to optimality than Group B/A) compared to the null, we found moderate evidence in favour of Reward Rate-Trial being closer to optimality than None-Time (compared to the null), and moderate evidence in favour of Reward Rate-Trial being closer to optimality than None-Trial (compared to the null). Most notably, when looking at a direct comparison between hypotheses that posited one group was closer to optimality than the other, we found moderate evidence in favour of Reward Rate-Trial being closer to optimality than each of the other three groups (compared to their respective inverses).

Experiment 3

Plots comparing actual threshold to optimal threshold for each group revealed the same two general trends as Experiments 1 and 2: that both groups were more cautious than optimality (much more so than Experiments 1 and 2, due to the optimal band reflecting a much more urgent strategy than in previous experiments), and that both groups became closer to optimality over blocks (though this was less the case for the Reward Rate-Time group; see Figure 3a). As with Experiments 1 and 2, a fixed trial group – in this case, None-Trial – appeared to come closest to optimality, though any differences between the groups appeared to be minor at best.

Quantitative results (see Figure 3b and Table 3) showed weak evidence for no difference between groups (Bayes factor [Effect vs Null] = 0.67), with even the direct comparison between hypotheses that posited one group was closer to optimality than the other showing no clear superiority for either group (Bayes factor [A vs B] = 0.79).

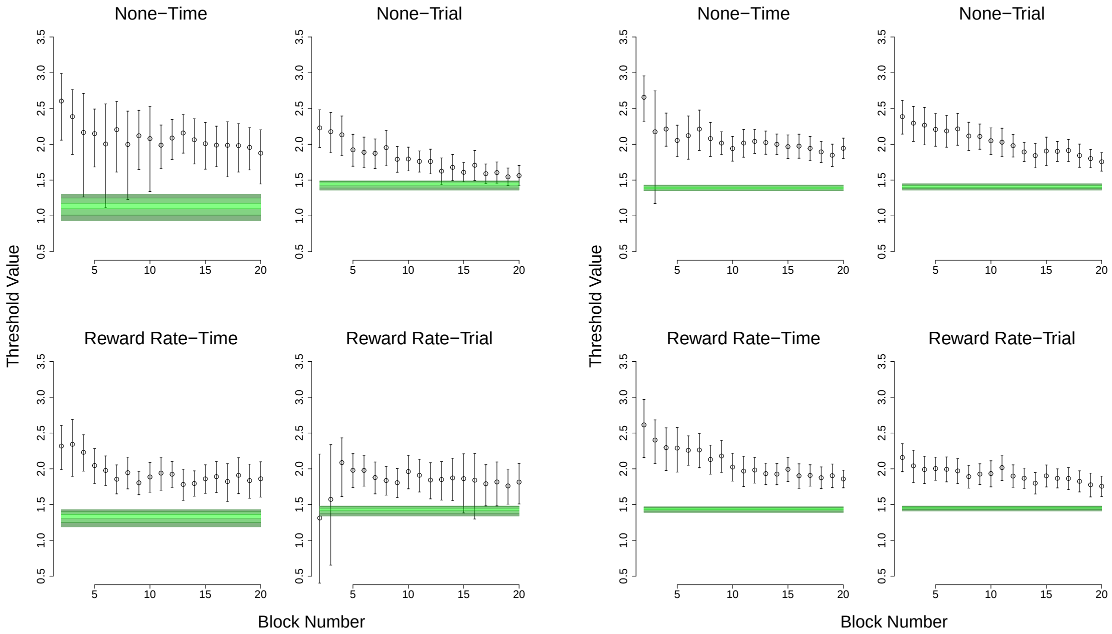

(a) Experiment 1 (b) Experiment 2

Figure 1. Actual threshold values for each condition, compared to their optimal threshold calculated using the Full Diffusion model. Circles represent median group level posterior threshold for each block (starting at block 2) with error bars showing the 95% quantiles. This is compared with the group averaged optimal threshold indicated by the coloured bands delineated by different shades of green. The lightest, centre band represents the 40 - 60% quantile, the middle shade represent the 20 - 40 % and 60 - 80% quantiles, and the darkest shade the 10 - 20% and 80 - 90% quantiles. Thresholds above the optimal band indicate cautious behaviour, whilst those below the band indicate more urgent behaviour.

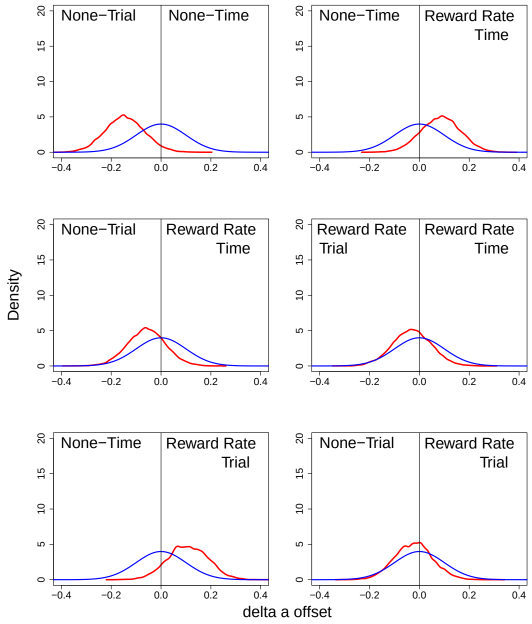

(a) Experiment 1

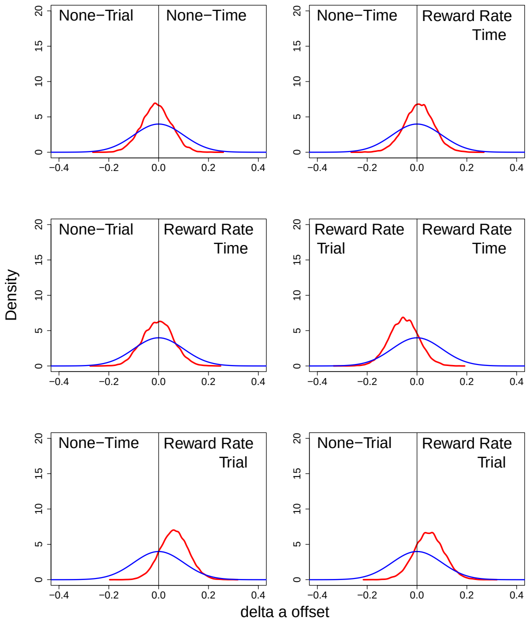

(b) Experiment 2

Figure 2. Prior-posterior distribution density plots of the group-level Δ_c parameter using the standard, normal prior distribution model. The prior distributions are in blue, and the posterior distributions are in red. Negative values indicate a superiority of the group named on the left side of the plot (i.e., above the negative numbers), whereas positive values indicate a superiority of the group named on the right side of the plot (i.e., above the positive numbers).

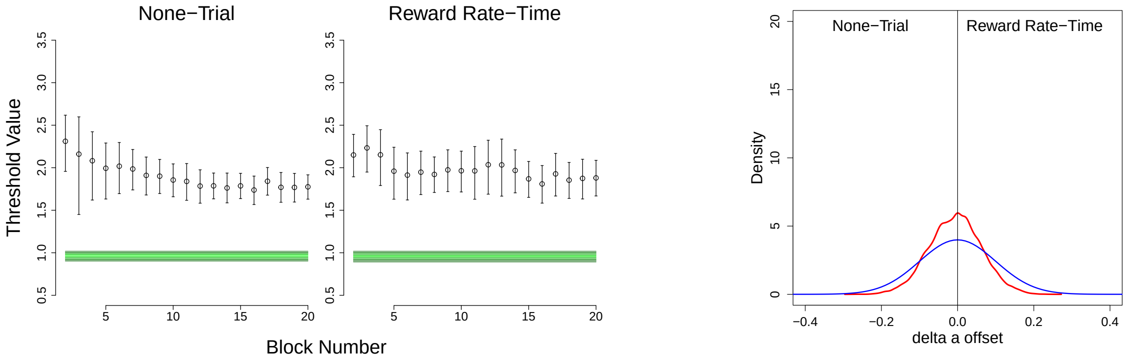

(a) Threshold Values (b) Density Plot

Figure 3. Experiment 3 comparisons of Reward Rate-Time/ None-Trial group combination.

Table 1: Savage Dickey Ratios for Experiment 1 using the Simple Diffusion Model, comparing how groups differ in their distance from optimality

	Prior Distribution						
Group Combination (A/B)	Normal	Positive Truncation		Negative Truncation		Ratio	
	Effect vs Null	A vs Null	A vs Null (adj)	B vs Null	B vs Null (adj)	A vs B	A vs B (adj)
None-Time / **None-Trial**	4.416	0.613	0.336	13.164	4.888	0.047	0.069
**Reward Rate-Time** / None-Time	1.436	4.523	1.851	0.783	0.448	5.776	4.136
Reward Rate-Time / **None-Trial** cf. SR2012	1.000	0.879	0.480	3.198	1.298	0.275	0.370
Reward Rate-Time / **Reward Rate-Trial** cf. EB2017	0.835	1.215	0.650	2.069	0.989	0.587	0.657
**Reward Rate-Trial** / None-Time	1.915	7.000	2.794	0.746	0.398	9.390	7.015
Reward Rate-Trial / **None-Trial**	0.759	1.214	0.644	1.884	0.909	0.644	0.676

Bold indicates which of the two groups is more likely to produce an optimal decision strategy; SR2012 – Starns & Ratcliff, 2012; EB2017 – Evans & Brown, 2017; Normal Distribution = effect vs. no effect;
Positive Truncation (directional) = effect in favour of group A vs. no effect; Negative Truncation (directional) = effect in favour of group B vs. no effect; Ratio = effect in favour of group A vs. effect in favour of group B;
adj = adjusted Bayes factors to compensate for densities very close to zero which may be higher than at zero.

Table 2: Savage-Dickey Ratios for Experiment 2 using the Simple Diffusion Model, comparing how groups differ in their distance from optimality

Group Combination (A/B)	Prior Distribution						
	Normal	Positive Truncation		Negative Truncation		Ratio	
	Effect vs Null	A vs Null	A vs Null (adj)	B vs Null	B vs Null (adj)	A vs B	A vs B (adj)
None-Time / **None-Trial**	0.602	1.060	0.542	1.297	0.639	0.817	0.848
**Reward Rate-Time** / None-Time	0.589	1.249	0.628	1.080	0.522	1.157	1.203
Reward Rate-Time / None-Trial cf. SR2012	0.638	1.210	0.613	1.168	0.600	1.036	1.021
Reward Rate-Time / **Reward Rate-Trial** cf. EB2017	0.868	0.628	0.364	2.605	1.149	0.241	0.317
**Reward Rate-Trial** / None-Time	0.996	3.519	1.259	0.558	0.315	6.306	3.997
**Reward Rate-Trial** / None-Trial	0.797	3.047	1.058	0.651	0.346	4.681	3.057

Bold indicates which of the two groups is more likely to produce an optimal decision strategy; SR2012 – Starns & Ratcliff, 2012; EB2017 – Evans & Brown, 2017; Normal Distribution = effect vs. no effect;
Positive Truncation (directional) = effect in favour of group A vs. no effect; Negative Truncation (directional) = effect in favour of group B vs. no effect; Ratio = effect in favour of group A vs. effect in favour of group B;
adj = adjusted Bayes factors to compensate for densities very close to zero which may be higher than at zero.

Table 3: Savage Dickey Ratios for Experiment 3 using the Full and Simple Diffusion models, comparing how groups differ in their distance from optimality.

Group Combination (A/B)	Prior Distribution						
	Normal	Positive Truncation		Negative Truncation		Ratio	
	Effect vs Null	A vs Null	A vs Null (adj)	B vs Null	B vs Null (adj)	A vs B	A vs B (adj)
**Reward Rate-Time** / None-Trial (FDM)	1.140	3.621	1.648	0.854	0.450	4.241	3.661
**Reward Rate-Time** / None-Trial (SDM)	0.670	1.401	0.652	1.773	0.640	0.790	1.019

FDM = Full Diffusion Model
SDM = Simple Diffusion Model

Individual Analysis

An analysis of individual performance was conducted for each experiment, comparing actual versus optimal threshold values, standardised using z scores, over blocks for each group (Evans, Bennett, & Brown, 2019). As the aim was to show that the individual-level estimates agree with the group-level estimates, the simple diffusion model was used so that estimates could be done analytically. Z scores presented in Figures 4 to 6, show that participants were mostly sub-optimally cautious, with a small few occasionally straying over to more risky decision-making. Similar to the main findings, there was some higher variability in the initial blocks, although subsequent consolidation to a thinner band is less distinct in comparison. Likewise, the overall trend is generally towards optimal decision-making as the task progresses, although in some cases the gradient is quite shallow. In summary, although individual results reveal a more nuanced picture, the average trend of the individual-level difference from optimality closely agrees with the equivalent analysis of group-level thresholds and optimal distributions.

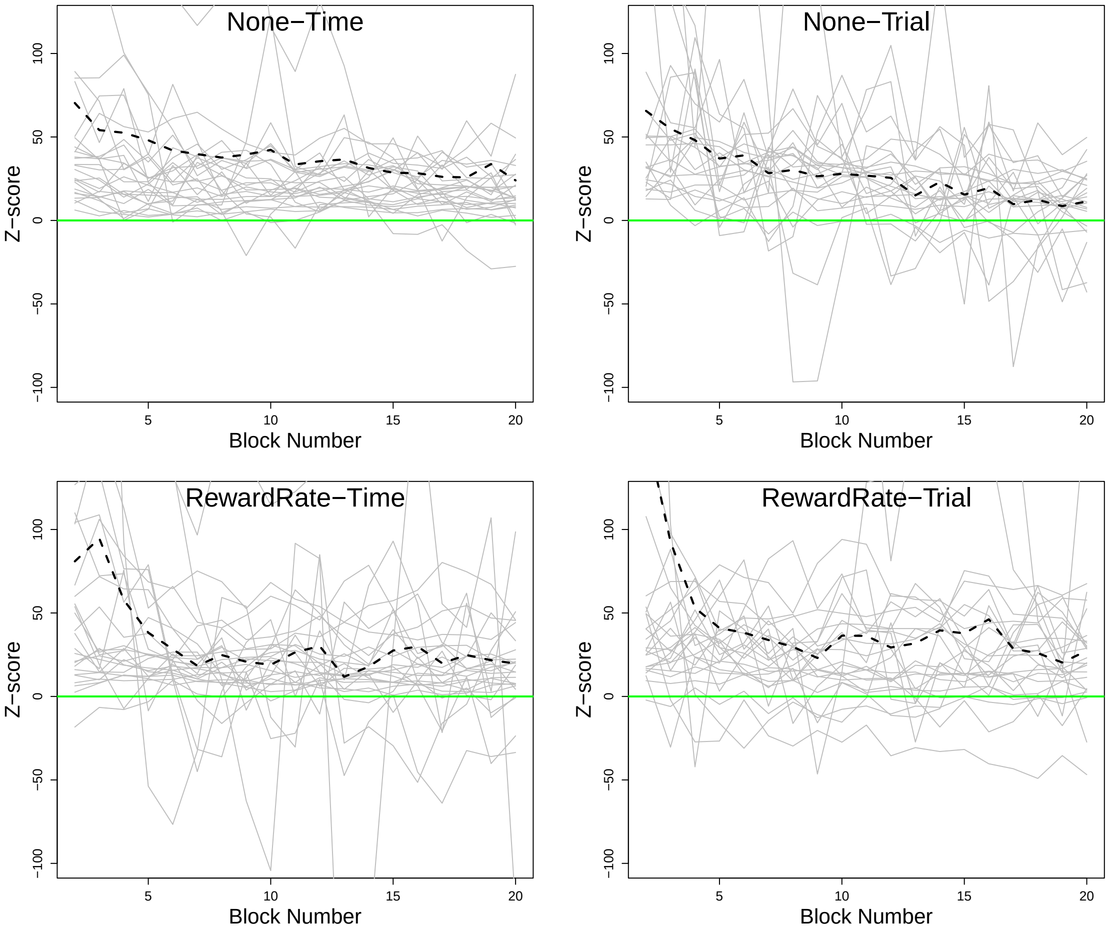

Figure 4. Z-score comparison Experiment 1. Grey lines indicate participant z-scores, black dash line indicates overall z-score trend.

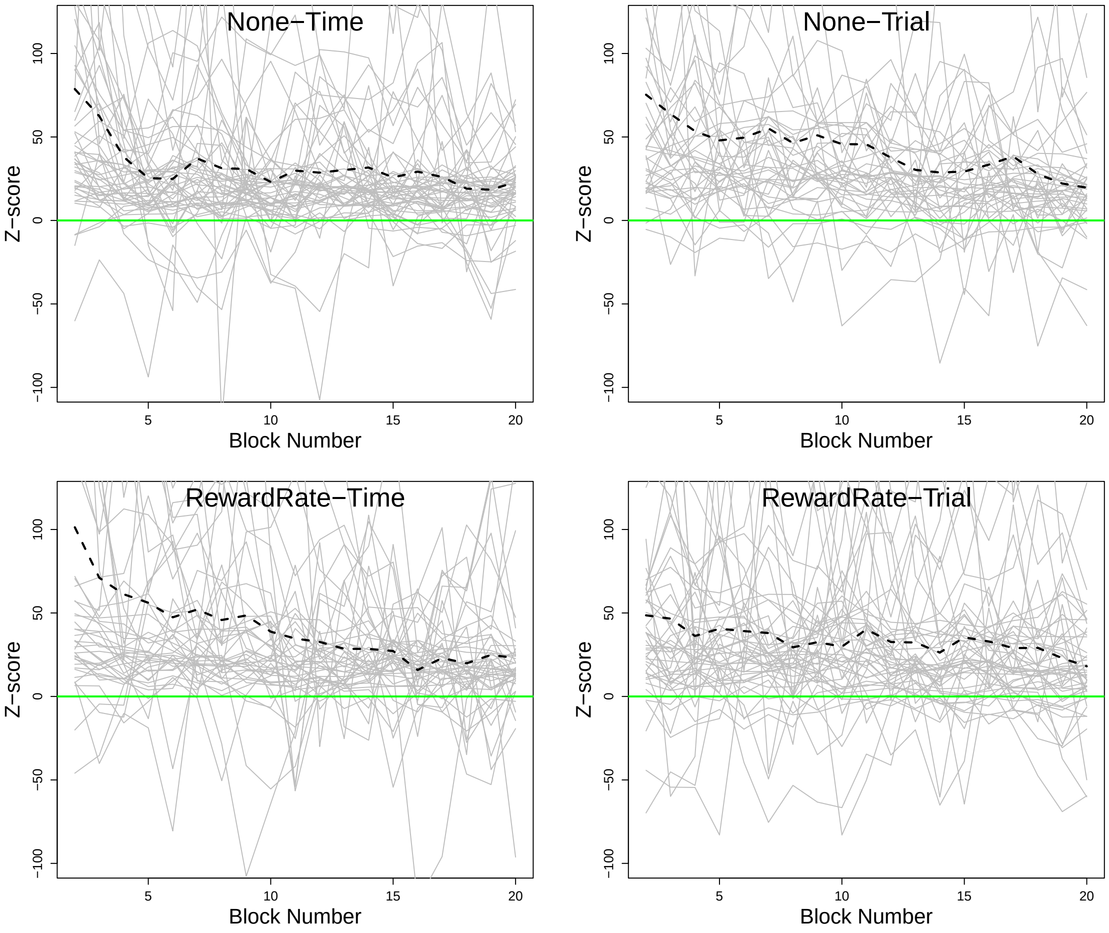

Figure 5. Z-score comparison Experiment 2. Grey lines indicate participant z-scores, black dash line indicates overall z-score trend.

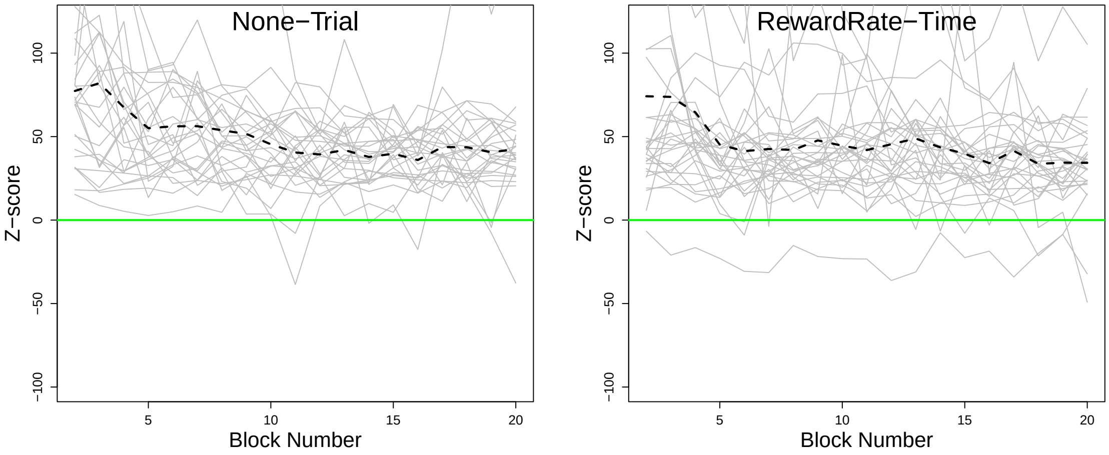

Figure 6. Z-score comparison Experiment 3. Grey lines indicate participant z-scores, black dash line indicates overall z-score trend.

Analysis of Reward Rate

An analysis of reward rate was undertaken to understand how groups differ on this basic metric, without it's inclusion in the optimality modelling. Reward rate was calculated as the accuracy rate divided by the average time spent on each trial; specifically, $\frac{PC}{MRT+ITI+FDT+(1-PC)*ET}$, where MRT reflects the mean response time, and PC reflects the accuracy, ITI reflects the inter-trial interval, FDT reflects the feedback display time, and ET reflects the additional timeout for error responses. For all experiments, ITI and FTD were the same (i.e., ITI=FTD= 0.5), whilst ET=1 for Exp1 and 2, and ET=0 for Exp3. For each experiment, an average reward rate was calculated for each participant, with either Bayesian 2 X 2 Anova (Exp 1,2) or independent-samples t-test (Exp 3) analysis completed at the group level. Analysis was completed using JASP statistical software (Version 0.19.2; JASP Team, 2024).

Note that in the tables below, "blkType" reflects how the experiment was administered with either a fixed amount of time per block ("Time") or a fixed number of trials per block ("Trial"). Likewise, "instrType" reflects insruction type, with either explicit instructions that encourage participants to pursue reward rate optimality ("Reward Rate") or typical instructions from the decision-making literature with no clear performance goal ("None").

Experiment 1

Results are summarised in Tables 4 to 6. Descriptive statistics (Table 4) show consistently higher mean reward rates in the "Trial" condition compared to "Time" across both "instrType" levels. Likewise, the main analysis (Table 5) highlights strong evidence for the effect of "blkType", with the model including "blkType" yielding the highest posterior probability ($P(M|data) = 0.542$) and Bayes factor ($\text{BF}_{10} = 4.080$), indicating that the "Trial" condition has a significantly greater effect than "Time". Conversely, the Averaged

Posterior Summary (Table 6) does not provide strong evidence to suggest that Trial produces a stronger effect than Time (or vice versa) within blkType. Both levels have small and statistically uncertain effects, as indicated by their overlapping 95% credible intervals. Similarly, the effect of "instrType" is negligible, as both its individual and interaction effects have low posterior probabilities, and their posterior means are close to zero with credible intervals including zero. The findings suggest that "blkType" is the primary driver of variability in reward rates, while "instrType" and its interaction with "blkType" do not meaningfully contribute.

Table 4: Descriptives - Experiment 1

						95% Credible Interval	
instrType	blkType	N	Mean	SD	SE	Lower	Upper
None	Time	27	0.279	0.101	0.020	0.239	0.319
	Trial	23	0.371	0.117	0.024	0.320	0.421
RewardRate	Time	26	0.331	0.120	0.023	0.283	0.380
	Trial	25	0.356	0.109	0.022	0.311	0.401

Table 5: Model Comparison - Experiment 1

Models	P(M)	$P(M \mid \text{data})$	BF_M	BF_{10}	error %
Null model	0.200	0.133	0.613	1.000	
blkType	0.200	0.542	4.739	4.080	0.007
instrType + blkType	0.200	0.166	0.795	1.248	0.511
instrType + blkType + instrType * blkType	0.200	0.117	0.529	0.878	1.435
instrType	0.200	0.042	0.176	0.318	0.022

Table 6: Model Averaged Posterior Summary - Experiment 1

				95% Credible Interval	
Variable	Level	Mean	SD	Lower	Upper
Intercept		0.364	0.004	0.350	0.377
instrType	None	−0.007	0.006	−0.020	0.006
	RewardRate	0.007	0.006	−0.006	0.020
blkType	Time	−0.004	0.006	−0.017	0.009
	Trial	0.004	0.006	−0.009	0.017
instrType * blkType	None-Time	0.003	0.006	−0.010	0.015
	None-Trial	−0.003	0.006	−0.016	0.010
	RewardRate-Time	−0.003	0.006	−0.016	0.010
	RewardRate-Trial	0.003	0.006	−0.010	0.015

Experiment 2

Results are summarised in Tables 7 to 9. Descriptive statistics (Table 7) show that across all conditions, reward rate (RR) is relatively stable, with means ranging from 0.355 to 0.379 and small standard errors (SE: 0.012–0.015). The main analysis (Table 8) shows that the null model has the highest posterior probability ($P(M|data) = 0.637$) and Bayes factor ($\mathrm{BF}_{10} = 1.000$), indicating no strong evidence for the inclusion of predictors (”instrType”, ”blkType”, or their interaction). The Model Averaged Posterior Summary in Table 9 show that both ”instrType” and ”blkType” effects are small with means close to zero and credible intervals spanning zero. The findings as a whole indicate no substantial differences in reward rates across the experimental conditions, with neither ”instrType” nor ”blkType” contributing to a meaningful explanation.

Experiment 3

Note that for the independent-samples t-test, ”instrType” and ”blkType” were collapsed to create two groupings - ”Reward Rate-Time” and ”None Trial”. The descriptive statistics in Table 10 show that the mean reward rates for the two groups, ”Reward Rate-

Table 7: Descriptives - Experiment 2

						95% Credible Interval	
instrType	blkType	N	Mean	SD	SE	Lower	Upper
None	Time	50	0.355	0.084	0.012	0.331	0.379
	Trial	41	0.357	0.086	0.013	0.330	0.384
RewardRate	Time	43	0.363	0.096	0.015	0.334	0.393
	Trial	48	0.379	0.094	0.013	0.351	0.406

Table 8: Model Comparison - Experiment 2

Models	P(M)	P(M—data)	BF_M	BF_{10}	error %
Null model	0.200	0.637	7.017	1.000	
instrType	0.200	0.187	0.919	0.293	0.040
blkType	0.200	0.131	0.603	0.206	0.052
instrType + blkType	0.200	0.037	0.152	0.057	0.632
instrType + blkType + instrType * blkType	0.200	0.009	0.035	0.014	1.533

Table 9: Model Averaged Posterior Summary - Experiment 2

				95% Credible Interval	
Variable	Level	Mean	SD	Lower	Upper
Intercept		0.364	0.004	0.350	0.377
instrType	None	−0.007	0.006	−0.020	0.006
	RewardRate	0.007	0.006	−0.006	0.020
blkType	Time	−0.004	0.006	−0.017	0.009
	Trial	0.004	0.006	−0.009	0.017
instrType * blkType	None - Time	0.003	0.006	−0.010	0.015
	None - Trial	−0.003	0.006	−0.016	0.010
	RewardRate - Time	−0.003	0.006	−0.016	0.010
	RewardRate - Trial	0.003	0.006	−0.010	0.015

Time" and "None Trial", are very similar. This is supported by the similarity in 95% credible intervals, which reinforces the conclusion of minimal differences in reward rates across conditions. Likewise, the Bayes factor in the main analysis (Table 11; $BF_{10} = 0.268$) indicates moderate evidence in favor of the null hypothesis, suggesting no substantial difference in reward rates between the "Reward Rate-Time" and "None Trial" groups. As such, there is little evidence to suggest that reward rates differ meaningfully between the two groups, as both descriptive statistics and Bayesian analysis support the null hypothesis.

Table 10: Descriptives - Experiment 3

					95% Credible Interval	
Group	N	Mean	SD	SE	Lower	Upper
None_Trial	28	0.400	0.087	0.016	0.366	0.434
RewardRate_Time	30	0.397	0.090	0.017	0.363	0.431

Table 11: Bayesian Independent Samples T-Test - Experiment 3

	BF_{10}	error %
Reward Rate	0.268	0.010

Discussion

Over the three experiments, there was very little difference between groups based on their reward rate, with only Experiment 1 indicating a difference between "Time" and "Trial" groups. Notably, differences favoured the "Trial" group in Experiment 1. Overall though, the lack of differences between groups based on reward rate aligns with the main finding that performance under fixed-trial compared to fixed-time conditions are at least similar to each other.

References

Boehm, U., Annis, J., Frank, M. J., Hawkins, G. E., Heathcote, A., Kellen, D., … Wagenmakers, E.-J. (2018). Estimating across-trial variability parameters of the diffusion decision model: Expert advice and recommendations. *Journal of Mathematical Psychology*, *87*, 46-75. doi: https://doi.org/10.1016/j.jmp.2018.09.004

Bogacz, R., Brown, E., Moehlis, J., Holmes, P., & Cohen, J. D. (2006). The physics of optimal decision making: a formal analysis of models of performance in two-alternative forced-choice tasks. *Psychological review*, *113*(4), 700.

Crüwell, S., Stefan, A. M., & Evans, N. J. (2019). Robust standards in cognitive science. *Computational Brain Behavior*, *2*(3-4), 255-265. doi: 10.1007/s42113-019-00049-8

Evans, N. J., Bennett, A. J., & Brown, S. D. (2019). Optimal or not; depends on the task. *Psychonomic Bulletin Review*, *26*(3), 1027-1034. doi: 10.3758/s13423-018-1536-4

Evans, N. J., Tillman, G., & Wagenmakers, E.-J. (2020). Systematic and random sources of variability in perceptual decision-making: Comment on Ratcliff, Voskuilen, and McKoon (2018). *Psychological Review*, *127*(5), 932-944. doi: 10.1037/rev0000192

JASP Team. (2024). *JASP (Version 0.19.2)[Computer software].* Retrieved from `https://jasp-stats.org/`

Lerche, V., & Voss, A. (2016). Model complexity in diffusion modeling: Benefits of making the model more parsimonious. *Frontiers in Psychology*, *7*(1324). doi: 10.3389/fpsyg.2016.01324

Ratcliff, R. (1978). A theory of memory retrieval. *Psychological review*, *85*(2), 59. doi: 10.1037/0033-295X.85.2.59

Ratcliff, R., & Rouder, J. N. (1998). Modeling response times for two-choice decisions. *Psychological Science*, *9*(5), 347-356. doi: 10.1111/1467-9280.00067

van Ravenzwaaij, D., Donkin, C., & Vandekerckhove, J. (2017). The ez diffusion model provides a powerful test of simple empirical effects. *Psychonomic Bulletin Review*, *24*(2), 547-556. doi: 10.3758/s13423-016-1081-y
